# Cancer-associated fibroblast-derived SDF-1 induces epithelial-mesenchymal transition of lung adenocarcinoma via CXCR4/β-catenin/PPARδ signalling

**DOI:** 10.1038/s41419-021-03509-x

**Published:** 2021-02-26

**Authors:** Yingyan Wang, Wen Lan, Mingxin Xu, Jing Song, Jun Mao, Chunyan Li, Xiaohui Du, Yunling Jiang, Encheng Li, Rui Zhang, Qi Wang

**Affiliations:** 1 Department of Respiratory Medicine, The Second Affiliated Hospital, Dalian Medical University, No. 467 Zhongshan Road, Dalian, 116023 Liaoning Province China; 2 Laboratory Center for Diagnostics, Dalian Medical University, No. 9 West Section Lvshun South Road, Dalian, 116044 Liaoning Province China; 3 Department of Respiratory Medicine, Ganzhou Municipal Hospital, No. 49 Dagong Road Zhanggong district, Ganzhou, 341000 Jiangxi Province China; 4 Department of Pathology, Dalian Medical University, No. 9 West Section Lvshun South Road, Dalian, 116044 Liaoning Province China; 5 Department of Gastroenterology, The First Affiliated Hospital, Dalian Medical University, No. 222 Zhongshan Road, Dalian, 116011 Liaoning Province China; 6 Department of Scientific Research Center, The Second Affiliated Hospital, Dalian Medical University, No. 467 Zhongshan Road, Dalian, 116023 Liaoning Province China

**Keywords:** Cancer microenvironment, Non-small-cell lung cancer

## Abstract

Cancer-associated fibroblasts (CAFs) contribute to tumour epithelial-mesenchymal transition (EMT) via interaction with cancer cells. However, the molecular mechanisms underlying tumour-promoting EMT of CAFs in lung adenocarcinoma (ADC) remain unclear. Here, we observed that CAFs isolated from lung ADC promoted EMT via production of stromal cell-derived factor-1 (SDF-1) in conditioned medium (CM). CAF-derived SDF-1 enhanced invasiveness and EMT by upregulating CXCR4, β-catenin, and PPARδ, while downregulating these proteins reversed the effect. Furthermore, RNAi-mediated *CXCR4* knockdown suppressed β-catenin and PPARδ expression, while β-catenin inhibition effectively downregulated PPARδ without affecting CXCR4; however, treatment with a PPARδ inhibitor did not inhibit CXCR4 or β-catenin expression. Additionally, pairwise analysis revealed that high expression of CXCR4, β-catenin, and PPARδ correlated positively with 75 human lung adenocarcinoma tissues, which was predictive of poor prognosis. Thus, targeting the CAF-derived, SDF-1-mediated CXCR4 β-catenin/ PPARδ cascade may serve as an effective targeted approach for lung cancer treatment.

## Introduction

Lung adenocarcinoma (ADC), a subtype of non-small cell lung cancer (NSCLC), is one of the most aggressive and fatal types of malignant tumour. Despite advancements in early detection diagnosis and therapeutic approaches for this disease, more than half of all lung ADC cases have a 5-year survival rate of 4% with advanced metastases^1^. Hence, better understanding of the metastatic mechanisms and identification of the associated signalling nodes or pathways will help to prevent metastasis and improve prognosis in patients with lung ADC. Epithelial-mesenchymal transition (EMT) is associated with the transformation of early tumours into aggressive malignant tumours^2–4^. When undergoing the process of EMT, cells acquire mesenchymal markers but lose both their polarity and epithelial markers, resulting in decreased cell-cell adhesion and increased mobility^5^. Indeed, EMT has been shown to be activated and to serve as an important event in lung ADC progression, invasion, and metastasis;^6,7^ however, the signalling networks underlying EMT remain obscure.

Cancer-associated fibroblasts (CAFs), the major components of the tumour stroma in the tumour microenvironment, play a key function in the malignant development of lung ADC^7–9^. CAFs are generally derived from activated fibroblasts within the tumour microenvironment. Compared to the latter, CAFs are characterised by overexpression of a specific subset of biomarkers, such as FAP-α, α-SMA, PDGFR-α/β, or S100A4, and neural-glial antigen^10,11^, depending on the tumour type. Generally, CAFs actively communicate with cancer cells mainly via the increased secretion of several factors, including cytokines and chemokines, TGF-β^12^, stromal cell-derived factor-1 (SDF-1)^13^, TNFα^14^, and exosomes^15,16^. Among them, SDF-l has been shown to be significantly upregulated in many cancer-related fibroblasts, including lung ADC-associated fibroblasts^17^. Furthermore, in breast cancer and colon cancer, aberrant production and signalling of SDF-1 has been reported to exert EMT-like effects via binding to its receptor, CXCR4 (seven-transmembrane G protein-coupled receptors) in the tumour cell membrane^18,19^. This aggregate triggers the activation of the core β-catenin protein in the canonical WNT/β-catenin signalling pathway and its sign transducers^20,21^. Thus, activation of the SDF-1/CXCR4/β-catenin pathway is important for carcinoma metastases and progression. Furthermore, PPARδ (peroxisome proliferator-activated receptor δ) is a known potential downstream effector of β-catenin^22–25^. PPARδ, a nuclear transcriptional receptor, is multifunctional, as it participates in glucose and lipid metabolism, inflammation, as well as cancer-associated biological processes, including EMT; its expression is upregulated in lung cancer^26,27^. However, the effects of PPARδ on lung ADC EMT have not been well addressed. Therefore, we aimed to elucidate the mechanism by which CAF enhances tumour invasion and EMT of lung ADC cells. In particular, we investigated the relationship between CAF and activation of the SDF-1/CXCR4/β-catenin/PPARδ signalling pathway within the context of lung cancer ADC.

To address this issue, CAFs were isolated from fresh lung ADC samples. We observed that CAFs-derived SDF-1 promoted tumour EMT by activation of the CXCR4/β-catenin/PPARδ pathway in lung cancer ADC. These results indicate that targeting SDF-1 or its downstream targets could serve as an effective targeted approach for lung cancer treatment.

## Materials and methods

### Separation and culture of cancer-associated fibroblast

**Fig. 1:**
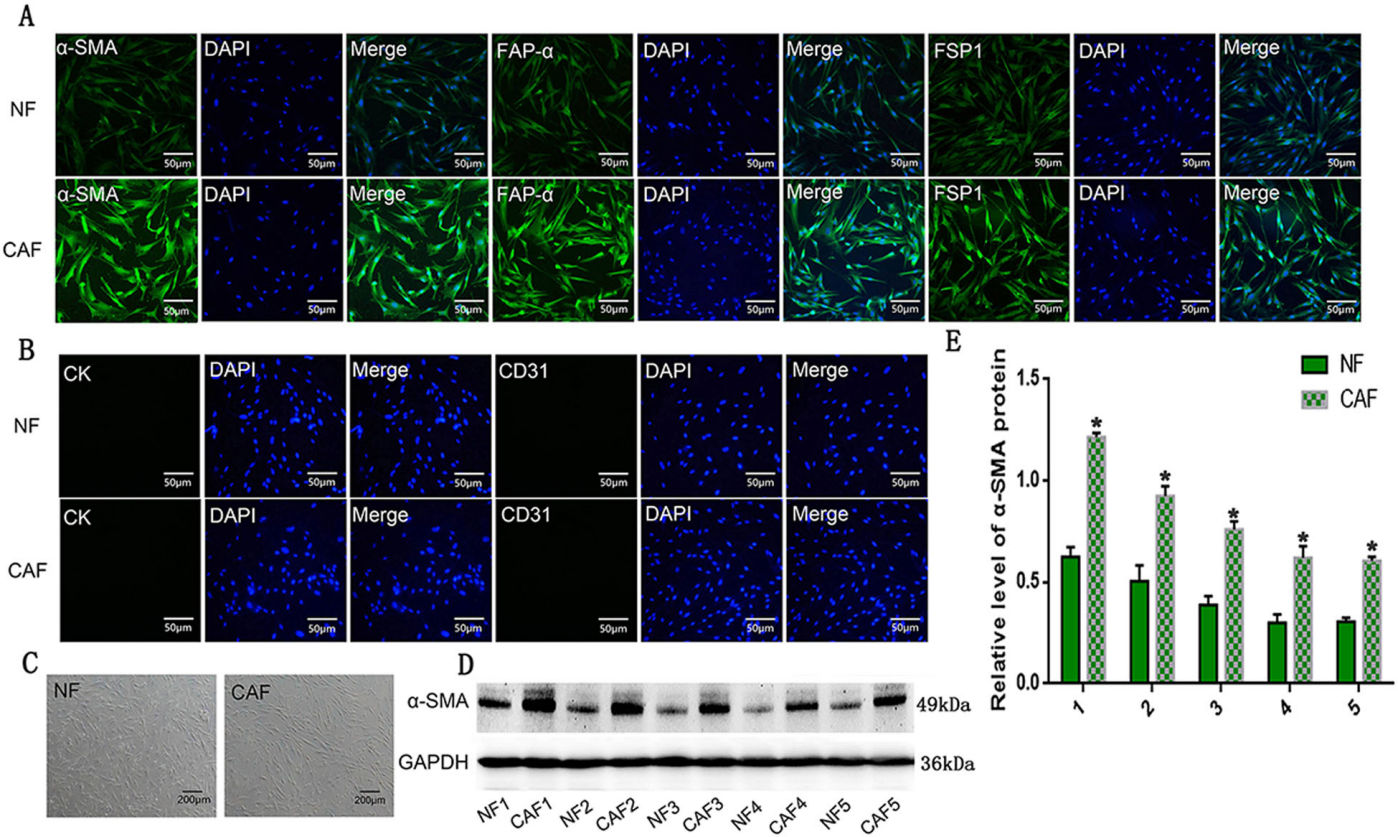
Characterisation of primary CAFs and NFs. **a**, **b** Immunofluorescent (IF) staining for the fibroblast markers (α-SMA, FAP-α and FSP1), the epithelial markers (CK) and the endothelial marker (CD31) in CAFs and NFs; scale bar: 50 μm. **c** Phase-contrast micrograph showing the shape of CAF and NF; scale bar: 200 μm. **d**, **e** Western blot analysis showed the expression of α-SMA in the CAF of seven samples. Data are representative images and numerical data are represented as the mean ± SD of each group of cells from three separate experiments. *p < 0.05.

Fresh lung cancer samples were collected from seven patients with pathologically diagnosed lung ADC. The patients had undergone surgical resection in 2017 at the Second Affiliated Hospital of Dalian Medical University (Liaoning, China). Consent to collect tissue samples and to conduct this study was obtained from all parties and stakeholders involved including the Dalian Medical University, the Human Research Ethics Committee of the Second Affiliated Hospital and all participants. We first cut the samples into blocks of approximately 1 to 2 mm in diameter. Secondly, we digested the tissues with trypsin and 0.5% collagenase before filtering them with a strainer. We subsequently isolated fibroblasts from the cell suspension. The isolation was completed with the aid of human anti-fibroblast microbeads following the producer’s guidelines (Miltenyi Biotec, Germany). The isolated cells were further cultured in F12K medium (Gibco, LifeTech, USA) supplemented 10% foetal bovine serum (FBS) (Gibco, Life Tech, USA) at 37 °C and 5% CO_2_. After 24 h, the first medium was changed. CAFs were identified by means of morphology and staining for α-SMA, FAP-α, FSP1, cytokeratin and CD31. (Fig. 1)

### Culture of lung cancer cell lines

Human lung adenocarcinoma H1299, A549, SPCA-1, PC9, NCI-H522, NCI-H1975, and BEAS-2B cells were purchased from the Chinese Academy Medical Sciences (Beijing, China). The cells were then cultured in RPMI-1640 medium supplemented with 10% foetal bovine serum at 37 °C and 5% CO_2_. These cells were identified by STR Genotyping at Shanghai Biowing Applied Biotechnology.

### Preparation of culture medium for cancer-associated fibroblasts and normal fibroblasts

CAFs were transferred to a 6-well plate at a density of 1.5×10^5^ cells/mL and cultured in F12K medium for 72 h. Next, the conditioned medium were collected and centrifuged at a speed of 300 g×10 min. Then cell debris was removed and ultimately the CAF-CM was obtained. Using the same procedure, the conditioned medium of NFs (NFs-CM) was collected.

### Indirect co-culture model of cancer-associated fibroblasts and lung cancer cells

The lung ADC cell lines, SPCA-1 and A549, were seeded respectively in six-well plates, and CAFs were cultured alone to the 0.4-mm polyester membrane of a 12-mm transwell insert (Corning, NewYork, NY, USA) at a density of 1.5 ×105 cells/mL After 24 h, the transwell insert was moved to the six-well plates containing the lung cancer cell cultures. In this way, indirect co-culture model of cancer-associated fibroblasts and lung cancer cells was realised.

### Immunofluorescence staining

The lung ADC cells were cultured on coverslips for 24 h and then fixed with 4% paraformaldehyde, permeabilized with 0.25% of Triton X-100 (Solarbio), and treated with 5% BSA. Subsequently, the cells were washed twice with PBS and treated for 24 h with antibodies against α-SMA (R&D Systems), fibroblast activation protein, fibroblast-specific protein 1, CD31, and cytokeratin (Abcam, Cambridge, Massachusetts, USA), as well as vimentin and E-cadherin(13-1700, Invitrogen). After being incubated for 24 h with FITC-conjugated secondary antibodies (1:100, Invitrogen), the cells were stained with 4′6-diamidino-2-phenylindole (DAPI) and imaged using the confocal microscope (Lecia, Solms, Germany). All experiments were carried out at least 3 independent replicates.

### Western blotting

Western blotting analysis was performed using the same procedure demonstrated previously^7^. Briefly, the radioimmunoprecipitation assay lysis buffer (Beyotime, China) was used to extract total cell protein, which was then quantified using the bicinchoninic acid protein assay kit (Beyotime, China) and resolved on a 6–12% sodium dodecyl sulphate-polyacrylamide gel, and then incubated with relevant primary antibodies. GAPDH and histone (ZSGBBIO, China) were used as loading controls. The following primary antibodies were utilised in this research: anti-PPARδ (Proteintech), anti-CXCR4 (Proteintech, China), anti-vimentin (Proteintech), anti-GAPDH (Proteintech), anti-β-catenin (Proteintech), anti-Slug (Proteintech) and anti-N-cadherin (Proteintech). We used GSK3787 (Abcam) and XAV-939 (Abcam, UK) as PPARδ and β-catenin inhibitors, respectively. The enhanced chemiluminescence system (Bio-Rad, Hercules, EDA USA) was used to detect protein expression levels and Scion imaging software was used to capture images.

### Invasion assay

Before invasion assay, the human anti-α-SMA (R&D Systems) neutralising antibody was added to the CAF-CM at 37 °C for 24 h. CAF-CM, NF-CM and CAF-CM + anti-α-SMA were co-cultured respectively with lung cancer cells for 48 h. Cells cultured in serum-free medium were used as a negative control. For invasion assay, 2.5×10^5^ lung cancer cells were inoculated into the upper chamber containing 8 μm holes (Millipore, GER), and covered them with Matrigel (Corning BioCoat, USA, 1:7.5), then 500 μL of 10% FBS-containing medium was introduced to the lower wells. After incubating for 48 h at 37 °C in the presence of 5% CO_2_, the cells attached to the filter’s upper surface were removed using a cotton swab. The remaining cells were then stained with 0.5% crystal violet and counted using a microscope (Leica, TCSSP5II) in five predetermined fields for each membrane at ×400 magnification.

### Enzyme-linked immunosorbent assay

The lung cancer cells, CAF and NFs were cultured as described previously. According to the manufacturer’s instructions, the supernatants of these cells were collected for SDF-1 detection using an human SDF-1 ELISA kit (eBioscience, San Diego, CA, USA), Briefly, supernatants or standard human SDF-1 were added on immunoassay plates precoated with the capture antibody anti-Human SDF-1 and incubated for 2 h. After being washed, the plates were incubated with biotin labelled SDF-1antibody for 2 h, then with streptavidin-HRP complex for 30 min. Then, tetramethylbenzidine (TMB) colour-substrate solution was added and incubated for 15 min. Each sample was read on a microplate reader (BioTek Instruments Inc., Winooski, VT, USA) at 450 nm.

### iTRAQ analysis

#### iTRAQ labelling

Trypsin gold (Promega, Madison, Wisconsin, USA) was used to digest a 100 μg aliquot of each sample at 37 °C for 16 h; The ratio of protein to trypsin is 30:1 (w/w). According to the method outlined by the manufacturer of the 8-plex iTRAQ reagent (Applied Biosystems),peptides were dried and reconstituted in 0.5 M TEAB (pH 8.5). Briefly, we melted a unit of iTRAQ reagent and then reconstituted it in 24 μL of isopropanol. Samples was marked with the following iTRAO tags: DP3-1,113; DP3-2115; DP3-3118; DP9-1,114; DP9-2117; DP9-3121. The peptides were labelled with isobaric tags and co-cultured at room temperature for 2 h. Finally, labelled the peptide fragment were collected and dried via vacuum centrifugation.

#### Strong cation exchange chromatography (SCX)

The LC-20AB HPLC pump system (Japan, Shimadzu, Kyoto, Japan) was used for SCX chromatography. The iTRAQ-labelled peptide mixture was reconstituted in 4 mL buffer A (25 mM NaH_2_PO_4_, 25% acetonitrile (ACN) containing pH 2.7), which was then loaded onto a 4.6 × 250 mm Ultremex SCX column containing 5 μm granules (Phenomenex). A peptide mixture was eluted by buffers A and B. The system was subjected to a gradient of buffer A for 10 min, 5–60% buffer B for 27 min, and 60–100% buffer B for 1 min, 100% buffer B for 1 min, buffer A for 10 min. To monitor elution, absorbance at 214 nm was measured, and fractions were collected every minute. Twenty fraction combination were collected from the eluted peptides, desalted on a Strata XC18 column (Phenomenex), and dried with a vacuum.

#### LC–MS/MS analysis

Each fraction was resuspended in solvent A (5% ACN, 0.1% FA) and loaded onto a 2 cm C18 trap column in the LC-20AD NanoHPLC system (Shimadzu, Kyoto, Japan). The peptide mixture was then eluted on a 10 cm analytical C18 column (inner diameter 75 μm) and separated at a total flow rate of 300 nL/min with a 35 min main gradient starting from 2 to 35% B (95%ACN, 0.1% FA), A triple TOF 5600 system (AB SCIEX, Concord, Ontario) was used for data acquisition. For TOF MS scans, the MS was operated with at least 30 000 FWHM of RP (reverse-phase). A survey scan was performed within 250 milliseconds to assist with IDA (information related to information acquisition). We were able to collect approximately 30 production scans based on the set threshold of 120 counts per second (counts/s) and with a 2+ to 5+ charge-state. The Q_2_ transmission window was set as 100 Da for 100%. Additionally, a collision energy setting of 35 ± 5 eV, combined with the rolling collision energy-adjusted by iTRAQ, was utilised to all precursor ions for collision-induced dissociation. Dynamic exclusion was set for 1/2 of peak width (15 s), and then the precursor was refreshed off the exclusion list.

#### Data analysis

Proteome was used to convert raw data files into MGF files. We used proteomics to convert the records and searched on the discovery application 1.2 (PD 1.2, Thermo), [5600 msconverter], MGF files, discoverer 1.2 (PD 1.2, Thermo) and [5600 msconverter] was explored. The Mascot search engine (Matrix Science, London, UK; version 2.3.02) containing 127 497 human sequences was used to identify the proteins. The database used to be received from Uniprot on June 17, 2015. The accepted mass of the intact peptide was set to 0.05 Da, and that of the fragment ion was set to 0.1 Da. A permit a misaligned reduce in the trypsin digest. Modifications included N-terminal Q, oxidation, and deamidation, while carbamoyl, iTRAQ8plex, iTRAQ8plex were fixed modifications. The charge states of the peptide was set to +2 and +3. To reduce the chance of protein misidentification, we only included peptides with a 95% confidence interval, and the estimated FDR fee of profitable identification used to be ≤1.04%. The identification of every protein required the detection of at least one unique peptide. Regarding protein quantification, a protein was required to incorporate at least two individual spectra. A *T*-test was carried out to decide the significance of the differences of each protein between the different samples. We applied the median ratio in Mascot to assign the weighted and standardised quantitative protein ratio. The ratio between the 3rd generation DPC-CM and the 9th generation DPC-CM was directly obtained using the protein abundance of any given protein. The following conditions are considered as the basis for differential expression of proteins: three consistent replicates; ratio of *p*-value <0.05; a fold change of at least two replicates is >1.2.

### Bioinformatics analysis

The public database STRING model 10.0 (http://string-db.org/) was used to construct the protein PPI network. Differentially expressed proteins were defined as a significant difference more than twice in triplicate analysis for lung cells with *p*-value ≤0.05 and the ratio value >2. The Cytoscape software program was used to display interactive networks and the Mode algorithm was applied to compute interconnected subgraphs for complex PPI networks. Finally, the “KEGGs” was used for pathway analysis.

### *CXCR4* knockdown using siRNA

The siRNA sequences specific for CXCR4 were: CXCR4–siRNA–1, 5′-CGGGAUACCUACCAUCCUA-3′; CXCR4–siRNA–2, 5′-CAGCUCUACUGAGAAGAAU-3′. According to the manufacturer’s instructions, cells were transfected with siRNA using Lipofectamine™ RNAiMAX reagent (Invitrogen). Briefly, cells were seeded into 6-well plates using opti-MEM supplemented with 10% FBS and were incubated with siRNA for 24–72 h.

### Luciferase reporter assay

Lenti TCF/LEF reporter (Cignal Lenti TCF/LEF Reporter (luc) Kit: CLS-018L, Qiagen) and Steady-Glo Luciferase Assay System (E2510, Promega) were commercially available. A luciferase assay was performed according to the manufacturer’s protocols; the values were normalised in relation to protein concentration.

### Quantitative real-time PCR analysis

According to the manufacturer’s instructions, 1 µg of total RNA was extracted from each sample with Trizol reagent (Invitrogen). cDNA was prepared using the Reverse Transcription System (Promega). The relative mRNA expression level was tested by quantitative real-time PCR with SYBR Premix (Takara Bio, RR420A) and a Thermo Fisher QS DX Real-time PCR system.

### Chromatin immunoprecipitations

ChIP was performed with anti-β-catenin antibody as described previously. Briefly, cells were fractionated and purified nuclei were sonicated to shear chromatin. Purified chromatin fragments were incubated with the anti-β-catenin antibody overnight. DNA fragments binding to the antibody were pulled down with Protein G magnetic beads and purified for RT-PCR assays. All experiments were performed at least three times and each experiment contained three technical replicates.

### Human tissue microarray

The lung adenocarcinoma tissue chip (HLug-Ade150Sur-02, Outdo Biotech, Shanghai, China) incorporates seventy-five pairs of surgical lung adenocarcinomas and adjacent non-tumour specimens. The specimen’s two tissue array blocks consisted of 40 males and 35 females, aged between 25 and 84 years, with a median age of 59.8 years. All cases were diagnosed and staged according to the 7^th^ edition International Union Against Cancer/American Joint Committee on Cancer TNM classification. A follow up on all patients (with the exception of 17 cases who had insufficient records) was conducted between 3 to 5 years after surgery. This protocol was approved by the Ethics Review Committee of the Second Hospital of Dalian Medical University and the study was performed according to ethical and safe research practices of human subjects or tissues. Informed consent was obtained from all patients.

### Immunohistochemistry (IHC)

The tissue array blocks were treated as following: fixed with formalin, incubated with xylene, dehydrated with graded ethanol solution, cultured with methyl alcohol including 3% hydrogen peroxide, soaked in a citrate buffer, and IHC staining using the streptavidin peroxidase IHC detection kit with antibodies specific for CXCR4, β-catenin, and PPARδ. The immunostained sections were then evaluated by two lung pathologists who were blinded to the study design. Each sample was assessed for CXCR4, β-catenin, and PPARδ expression according to stain depth (negative stain: zero points, susceptible stain: 1 point, medium stain: two points, rich stain: 3 points). The scores were calculated by way of multiplying by way of a number—the percentage of stained cells. Score ≥6 point is defined as high expression, otherwise as low expression.

### Statistical analyses

Student’s *t*-test and Analysis of variance (ANOVA) were used to calculate the distinction between the test and control samples. Pearson’s chi-square analysis was used to assess the relationship between protein expression and categorical variables. A survival curve was constructed using the Kaplan–Meier method. Furthermore, log-rank analysis was performed to analyse the OS of lung ADC sufferers with unique clinicopathological features. SPSS was used for all statistical analyses. A *p*-value <0.05 was deemed statistically significant. All experiments were repeated a minimum of three time, and the mean values and standard deviations were calculated.

## Results

### Characterisation of primary cancer-associated fibroblasts and normal fibroblasts

To understand the function of CAFs in lung ADC, we isolated seven pairs of CAFs and normal fibroblasts (NFs) from fresh lung ADC tissue specimens and each pair of CAFs and NFs was collected from the same patient. Phase-contrast microscopy revealed the typical spindle-like features of fibroblasts in CAF and NFs (Fig. 1c), with the morphology of CAFs and NFs not differing significantly. To ensure the samples were not contaminated with other cell types and to identify the fibroblastic phenotype within the CAF and NF cell populations, expression of the epithelial cell marker, cytokeratin (CK), endothelial cells marker, CD31, and mesenchymal cell markers, FSP1, FAP-α, and α-SMA, were assessed. Immunofluorescence (IF) staining showed that CAF and NFs were positive for a-SMA, FAP-α, and FSP1 and negative for CK and CD31, indicating that the primary cultured cells were uniformly composed of fibroblasts and were not contaminated by other cell types. To characterise CAF and NF, we evaluated the expression of mesenchymal cell markers on each pair of CAF and NF from seven patients. Compared to NFs, the CAFs of five out of seven samples showed higher FSP1, FAP-α, and α-SMA levels by IF staining (Fig. 1a, b). Western blot analysis confirmed the increased expression of α-SMA in these five CAFs, indicating that they were once activated fibroblasts (Fig. 1d, e). These results suggest that a large proportion of the cultured fibroblasts from lung ADC specimens function as CAFs and NFs.

### Cancer-associated fibroblasts induce epithelial-mesenchymal transition in lung adenocarcinoma cells

**Fig. 2:**
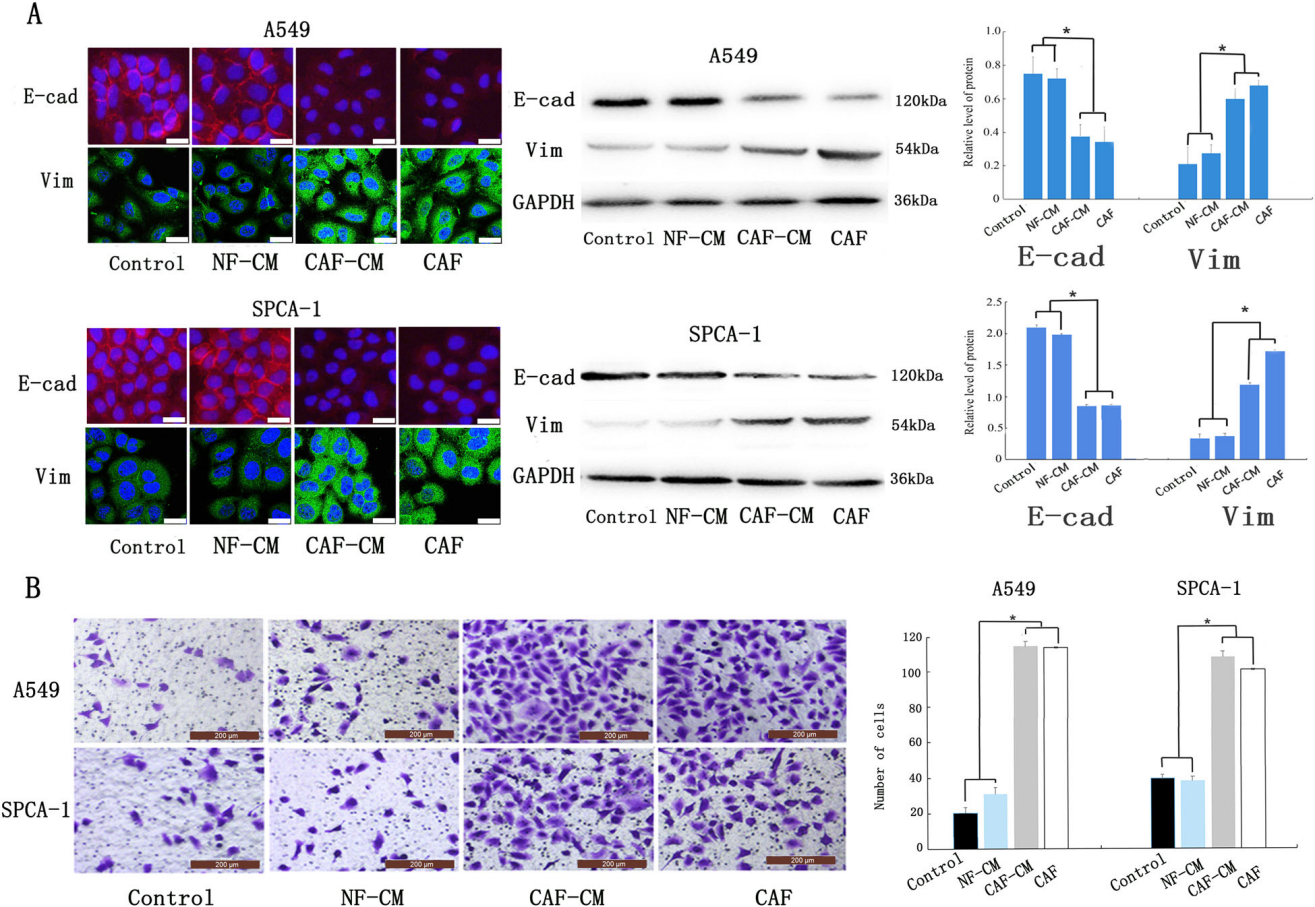
CAF-CM induces EMT in lung ADC cells. **a** Expression of epithelial marker E-cadherin and mesenchymal marker vimentin via western blotting analysis and immunofluorescent staining; scale bar: 25 μm. **b** Transwell assay examining the invasiveness of cells; scale bar: 200 μm. Data are representative images and numerical data are represented as the mean ± SD of each group of cells from three separate experiments. *p < 0.05.

To determine whether CAFs directly regulate EMT in a paracrine manner in vivo, the CAFs, CAF-conditioned media (CAF-CM), and their corresponding NF-conditioned media (NF-CM) were collected and used to culture A549 and SPCA-1 cell lines; DMEM was used as the control. After 48 houes, EMT-related proteins were detected using IF staining and western blotting. In both cell lines, the fluorescence intensity of vimentin in most lung cancer cells co-cultured with CAF-CM or CAF was higher than that co-cultured with NF-CM and control group. In contrast, E-cadherin fluorescence was decreased in the cells co-cultured with CAF-CM or CAF group compared to that co-cultured with NF-CM group and control group (Fig. 2a). Western blotting confirmed that E-cadherin expression was lower in cells co-cultured CAF-CM or CAF compared to these co-cultured with NF-CM and control group; alternatively, vimentin expression was upregulated in lung ADC cells co-cultured with CAF-CM or CAF compared to the NF-CM group and control group (Fig. 2b). Also, a Transwell assay shows that CAF-CM and CAF affected the invasiveness of lung ADC cells more than NF-CM (Fig. 2c). Results indicated that both CAFs and CAF-CM enhanced the invasiveness of lung ADC cells, and that significant differences in protein expression and invasiveness were not observed in the two groups. These results indicate that CAFs and CAF-CM both effectively regulate the EMT of lung ADC cells, suggesting that CAFs regulate lung cancer primarily via paracrine signalling.

### Cancer-associated fibroblasts regulate epithelial-mesenchymal transition in lung adenocarcinoma cells via paracrine secretion of SDF-1

**Fig. 3:**
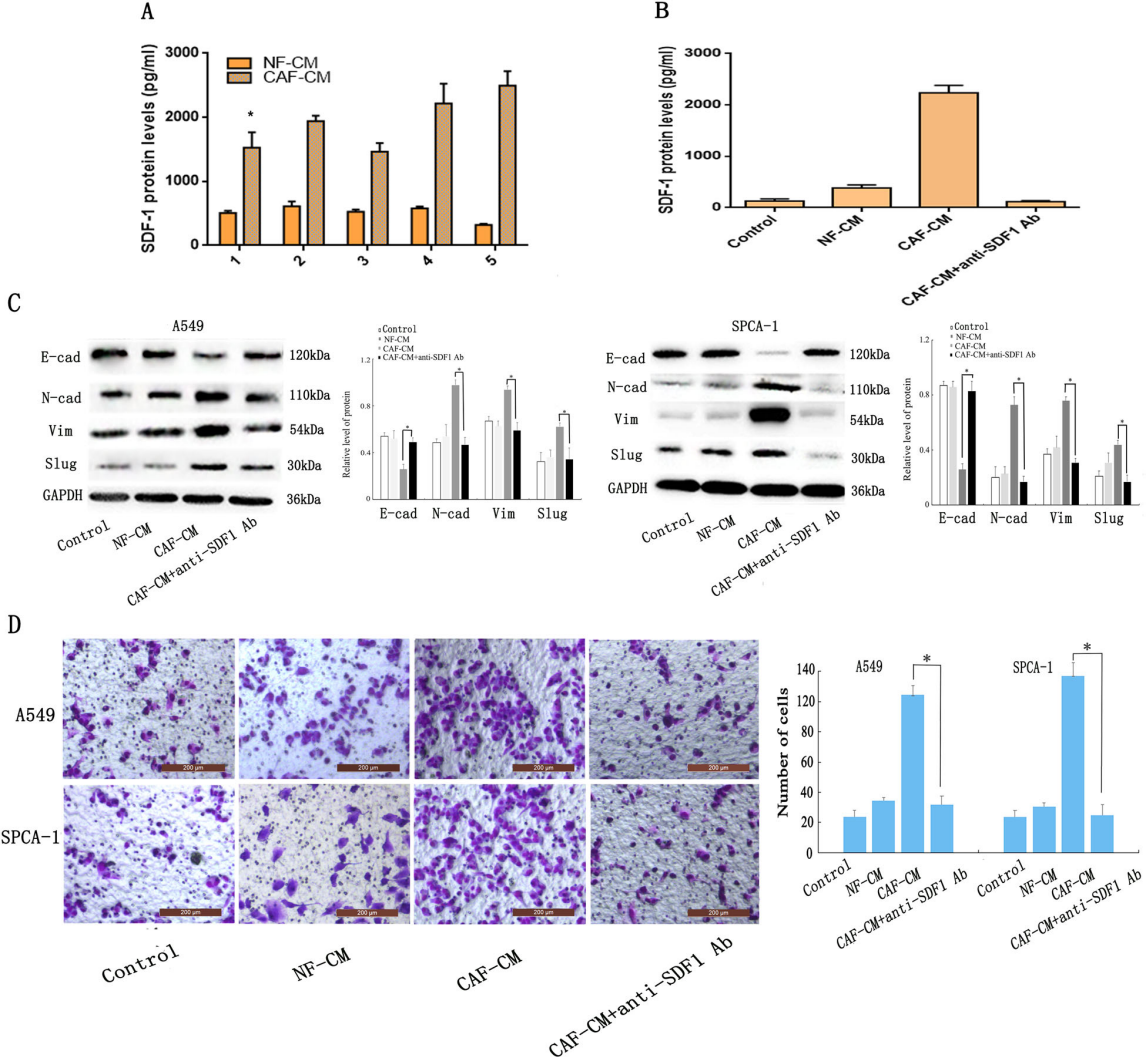
SDF-1 triggers CAF-induced EMT and invasion in lung cancer cells. **a**, **b** SDF-1 protein levels in the conditioned medium quantified by ELISA. **a** Five groups of CAF and corresponding NF cultured separately for 72 h. **b** Lung cancers cells incubated with DMEM, NF-CM, CAF-CM, and CAF-CM + SDF-1 neutralising antibody for 24 h. **c** Western blotting analysis of epithelial and mesenchymal marker expression. **d** Transwell assay examining the invasiveness of cells; scale bar: 200μm.The error bars represent suggested the standard errors of the mean of the three individual experiments. *p < 0.05.

As SDF-1 is a prominent EMT-inducer secreted by CAFs to CAF-CM, we determined SDF-1 protein expression in all five sets of CAF-CM and the matched NF-CM using enzyme-linked immunosorbent assay (ELISA). Results indicated higher levels of SDF-1 in the CAF1-CM (2.5-fold), CAF2-CM (2.8-fold), CAF3-CM (2.4-fold), CAF4-CM (3.4-fold), and CAF5-CM (6.2-fold) than in the corresponding NF-CM (Fig. 3a). To further investigate whether SDF-1 is involved in CAF-mediated EMT, we added SDF-1 neutralising antibody to A549 and SPCA1 cells cultured in CAF-CM (Fig. 3b), and analysed the invasiveness of EMT-related proteins in the lungs using a Transwell system and western blotting. Treatment with the SDF-1 neutralising antibody prevented the impact of CAF-CM on cell invasiveness (Fig. 3c, d), and increased E-cadherin expression in contrast to the CAF-CM group, whereas the protein levels of vimentin, N-cadherin, and Slug were decreased in both cell lines. Taken together, these results suggest that the impact of CAF on EMT was due to the presence of SDF-1 in the CM of CAFs.

### Analysing the mechanism underlying lung adenocarcinoma epithelial-mesenchymal transition induced by cancer-associated fibroblast-secreted SDF -1 using proteomics and bioinformatics

**Fig. 4:**
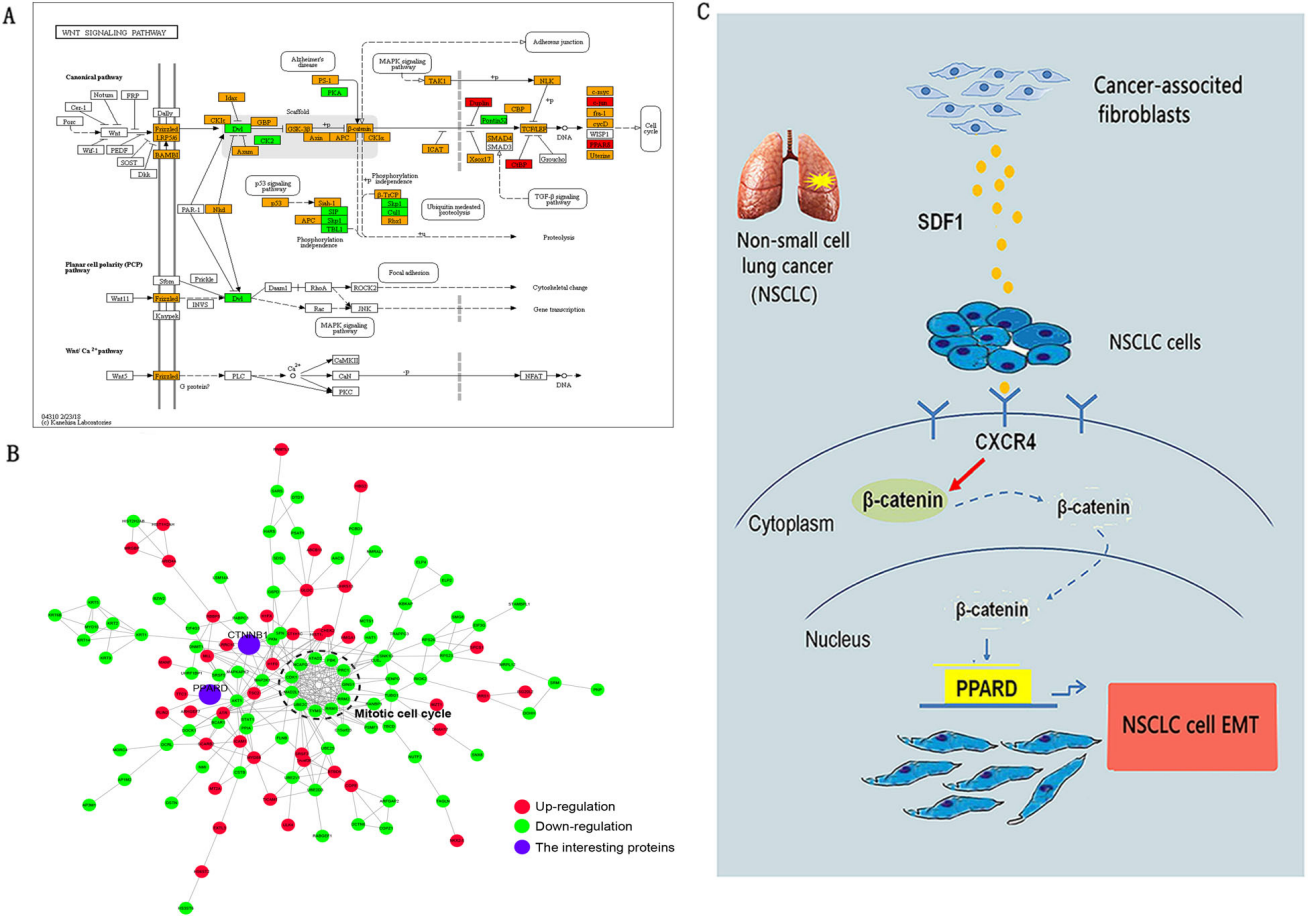
Analysis of the mechanism of lung ADC EMT induced by CAF-secreted SDF-1 using proteomics and bioinformatics. **a** Kyoto Encyclopedia of Genes and Genome (KEGG) pathways of different proteins related to β-catenin in EMT-A549 cells. **b** Molecular model map of differential genes related to the β-catenin pathway in EMT-A549 cells. **c** Schematic diagram showing the potential mechanism by which CAF-secreted SDF-1 mediates EMT in lung ADC cells.

Numerous studies have shown that SDF-1/CXCR4/β-catenin signalling is significant in tumour metastasis^28,29^. Therefore, we investigated whether the effect of SDF-1/CXCR4/β-catenin on EMT is also involved in the metastasis of lung ADC. To analyse the events downstream of β-catenin signalling during EMT, we treated A549 cells individually with CAF-CM or NF-CM for 48 h. We then detected cellular proteins using isobaric tags for relative and absolute quantification (iTRAQ) and compared the protein expression profiles of these cells using proteomic analysis. Table 1 provides a list of proteins that were upregulated in response to CAF-CM treatment. Our focus was on PPARδ, as it was upregulated 9−10 fold upon CAF-CM treatment, and was found to be a downstream effector of β-catenin in the “Cell Cycle” pathway of Kyoto Encyclopedia of Genes and Genome (KEGG) analysis (Fig. 4a) and protein-protein interaction (PPI) network (Fig. 4b). In addition, PPARδ, as a nuclear transcriptional receptor, is upregulated in several human cancers^30–33^, including lung cancer^27^, in which its expression is associated with metastasis^32^. In summary, we showed that SDF-1 secreted by CAF might act via the CXCR4/β-catenin/PPARδ signalling pathway to regulate lung cancer EMT (Fig. 4c).

**Table 1:** Differentially expressed downstream target genes of β*-*catenin before and after EMT in the lung ADC cell line A549.

Gene name	Protein name	Ratio (A549EMT/A549)
SNAI1	Zinc finger protein SNAI1	2.05
VIM	Vimentin	1.58
PPARD	PPAR-delta	9.39
JUN	Transcription factor AP-1	3.45
FN1	Fibronectin	2.86

### Cancer-associated fibroblast-secreted SDF-1 induces epithelial-mesenchymal transition of lung adenocarcinoma cells by upregulating the expression of CXCR4, β-catenin, and PPARδ

**Fig. 5:**
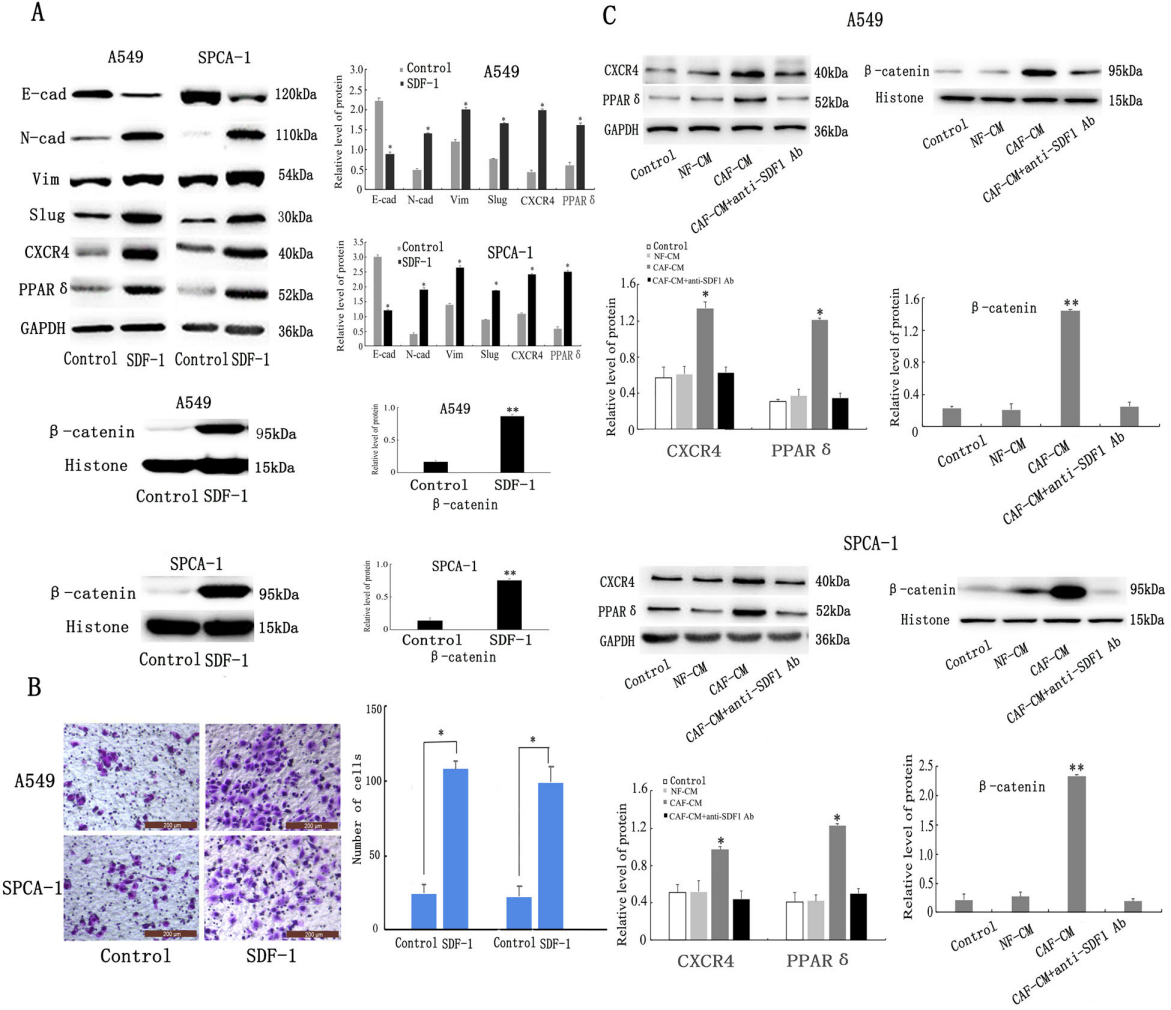
SDF-1 secreted by CAF induces EMT of lung ADC cells by upregulating the expression of CXCR4, β-catenin and PPARδ. **a** Western blot analysis showed that in the presence of exogenous SDF-1, CXCR4, β-catenin, PPARδ, vimentin, N-cadherin, and Slug were all upregulated, while E-cadherin was downregulated. **b** Transwell analysis showed that exogenous SDF-1 increased cell invasiveness; scale bar: 200 μm. **c** Western blotting showed that CXCR4, β-catenin and PPARδ were downregulated in cells cultured with CAF-CM + SDF-1 neutralising antibody. Data are representative images and numerical data are represented as the mean ± SD of each group of cells from three separate experiments. *p < 0.05; **p < 0.01.

To examine the potential features of SDF-1, we first investigated the effect of SDF-1 on signal transduction in lung ADC cells. To this end, we added SDF-1 to the culture medium of A549 and SPCA-1 cells and evaluated CXCR4, β-catenin, PPARδ, and EMT-related proteins using western blotting (Fig. 5a). As expected, the two types of lung cancer cells cultured with SDF-1 showed higher levels of CXCR4, β-catenin, PPARδ, vimentin, Slug and N-cadherin, and lower levels of, E-cadherin compared to cells not treated with SDF-1. Moreover, the Transwell assay (Fig. 5b) showed that cells cultured with SDF-1 were more invasive than those cultured without SDF-1. These results indicate that exogenous SDF-1 can induce EMT by upregulating the expression of CXCR4, β-catenin, and PPARδ. To verify whether CXCR4, β-catenin, and PPARδ could be regulated by SDF-1 in CAF-CM, we used the neutralising SDF-1 antibody to block SDF-1 signalling in the A549 and SPCA-1 cells. The results of western blotting and Transwell assay showed that treatment with the neutralising SDF-1 antibody decreased the CAF-CM-induced expression of CXCR4, β-catenin, and PPARδ (Fig. 5c) and blocked the effect of EMT on both cells (Fig. 3d), suggesting that SDF-1, CXCR4, β-catenin, and PPARδ are involved in CAF-CM-induced EMT in lung ADC cells.

### SDF-1 induces epithelial-mesenchymal transition of lung adenocarcinoma cells via CXCR4/β-catenin/ PPARδ signalling

**Fig. 6:**
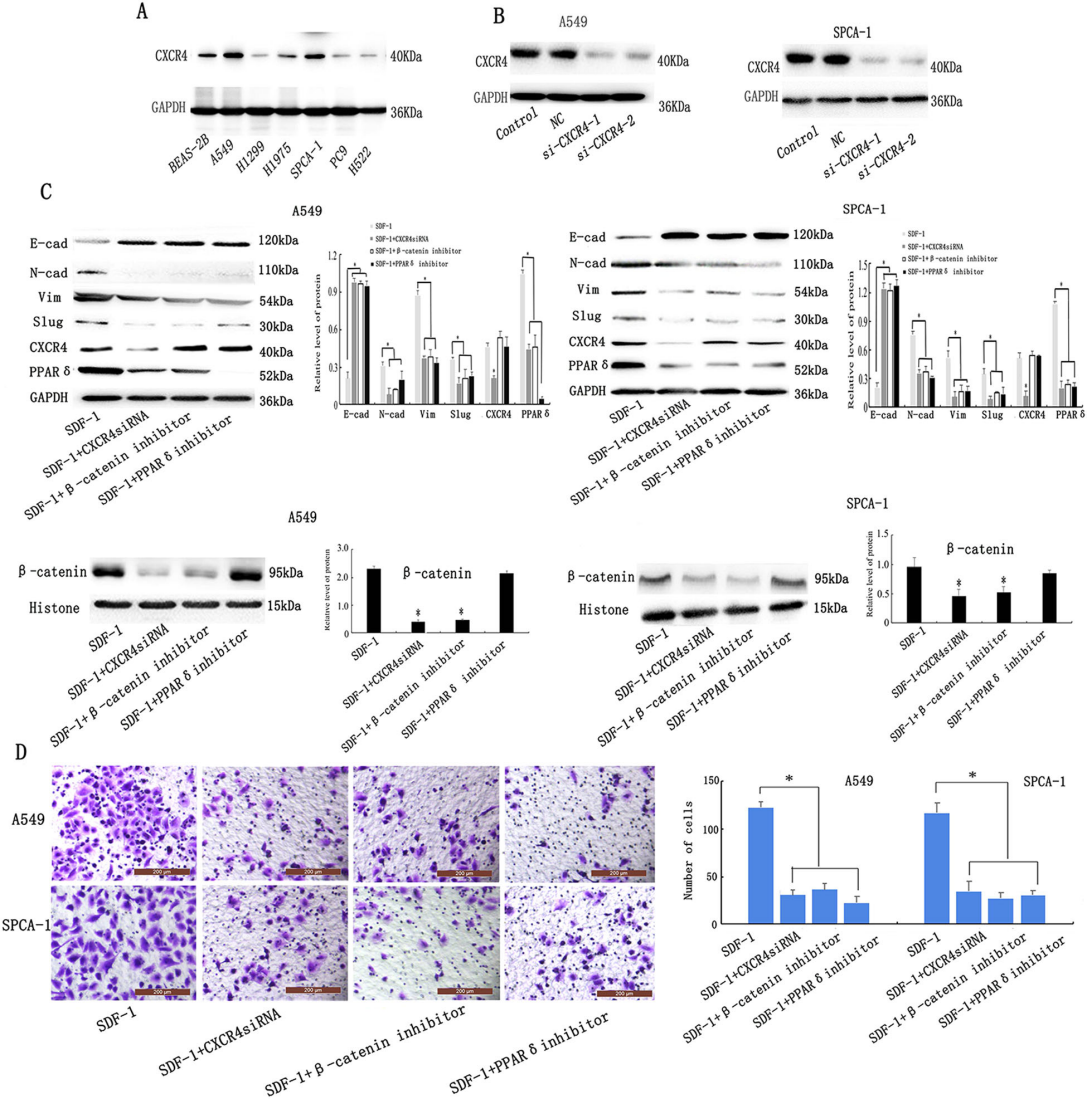
SDF-1 induces EMT of lung ADC cells via CXCR4/β-catenin/PPARδ signalling. **a**, **b** The expression of CXCR4 expression was detected using western blotting in several lung ADC cell lines. Compared with control cells (**b**), higher CXCR4 expression was observed in A549 and SPCA-1 cells (**a**), and lower CXCR4 expression was observed in *CXCR4* knockdown cells. **c** Western blotting was used to detect CXCR4, β-catenin, PPARδ and EMT-related proteins (vimentin, N-cadherin, Slug, and E-cadherin) in A549 and SPCA-1 cells. **d** Transwell analysis showed that *CXCR4* knockdown, or inhibition of β-catenin and PPARδ eliminated the invasion induced by SDF-1; scale bar: 200 μm. Data are representative images and numerical data are represented as the mean ± SD of each group of cells from three separate experiments. *p < 0.05.

We investigated whether SDF-1 binding to CXCR4 induced β-catenin and PPARδ and caused EMT in lung ADC cells. First, we identified which cancer cell lines adequately express CXCR4 (Fig. 6a) and ultimately selected A549 and SPCA-1, which were then transfected with CXCR4 siRNA. The knockdown efficiency of the siRNA was assessed by determining CXCR4 expression in both cell types (Fig. 6b). Cells with *CXCR4* knockdown were then exposed to SDF-1 for 48 h prior to conducting western blot and Transwell analysis. Following SDF-1 treatment, CXCR4 knockdown reversed the impact of SDF-1-induced EMT and inhibited β-catenin and PPARδ (Fig. 6c), which was confirmed using Transwell analysis (Fig. 6d). These outcomes suggest that CXCR4-bound SDF-1 regulates β-catenin and PPARδ expression, as well as EMT in A549 and SPCA-1 cells.

The SDF-1/CXCR4 axis is associated with activation of β-catenin signalling in the bladder and colorectal cancers^20,27^. We, therefore, sought to determine whether these pathways affect lung ADC and result in PPARδ expression in A549 and SPCA-1 cells. We first evaluated the effect of pre-treating A549 and SPCA-1 cells with 1 μM XAV-939 (a unique β-catenin inhibitor) for 24 h before SDF-1 treatment. Compared to cells treated with SDF-1 alone, those exposed to both SDF-1 and XAV-939 exhibited significantly reduced invasive ability in Transwell assays (Fig. 6d). Moreover, altered EMT-related protein expression was also observed in these groups, such as downregulated expression of vimentin, N-cadherin, and Slug, and upregulated expression of E-cadherin (Fig. 6c). These results demonstrate that the β-catenin signalling pathway is required for EMT in A549 and SPCA-1 cells. Furthermore, compared to that in control group, XAV-939 significantly downregulated the expression of PPARδ, while CXCR4 expression was not affected, irrespective of whether the cells were treated with or without SDF-1 (Fig. 6c). Therefore, inhibition of β-catenin expression appears to reverse the effect of SDF-1-mediated EMT and downregulates the expression of PPARδ, without affecting CXCR4 expression, indicating that PPARδ is regulated as the downstream player of the β-catenin signalling pathway, while CXCR4, which is upstream of β-catenin, is not regulated.

In addition, β-catenin localisation and its activity were assessed using immunofluorescence and Luciferase reporter assay, respectively. We observed that β-catenin translocated from membrane to nucleus in cells cultured with CAF-CM, and these cells exhibited enhanced β-catenin-TCF transcriptional activity, while CXCR4 knockdown and the SDF-1 neutralising antibody inhibited CAF-CM-induced β-catenin mis-localisation and activity, indicating that β-catenin was activated by the SDF-1/CXCR4 axis in lung A549 and SPCA-1 cells. (Supplementary Fig. 1).

To verify the effect of PPARδ on the signalling pathway and EMT induced by SDF-1, cells were pre-treated with 1 μM GSK3787, a specific PPARδ inhibitor, for 24 h before SDF-1 treatment. Cells treated with GSK3787 did not respond to SDF-1 stimulation (Fig. 6c, d). Furthermore, the expression of the EMT-related proteins (vimentin, N-cadherin, Slug, and E-cadherin) did not change when PPARδ was inhibited in lung ADC cells. Similar to CXCR4 and β-catenin, PPARδ inhibitor also blocked the EMT-promoting effect of SDF-1 on lung cancer cells. In addition, PPARδ expression was markedly antagonised by *CXCR4* knockdown and by addition of the β-catenin inhibitor, whereas the PPARδ inhibitor GSK3787 did not inhibit CXCR4 or β-catenin expression. Hence, inhibition of PPARδ expression reversed the effect of SDF-1-mediated EMT, although the expression of β-catenin and CXCR4 was not affected, suggesting that PPARδ acts downstream of CXCR4 and β-catenin in the predicted signalling pathway. Meanwhile, the mRNA expression levels of PPARδ were measured by qRT-PCR and presented a trend similar to that shown by western blot analysis (Supplementary Fig. 2). To analysis the recruitment of β-catenin on PPARδ gene promoter, we performed ChIP analysis, showing that active β-catenin can bind to the PPARδ promoter (Supplementary Fig. 3)

Taken together, we concluded that SDF-1 binding to CXCR4 activates the β-catenin/PPARδ pathway in A549 and SPCA1 cells.

### High expression of CXCR4, β-catenin, and PPARδ correlates positively and is associated with poor prognosis of patients with lung adenocarcinoma

**Fig. 7:**
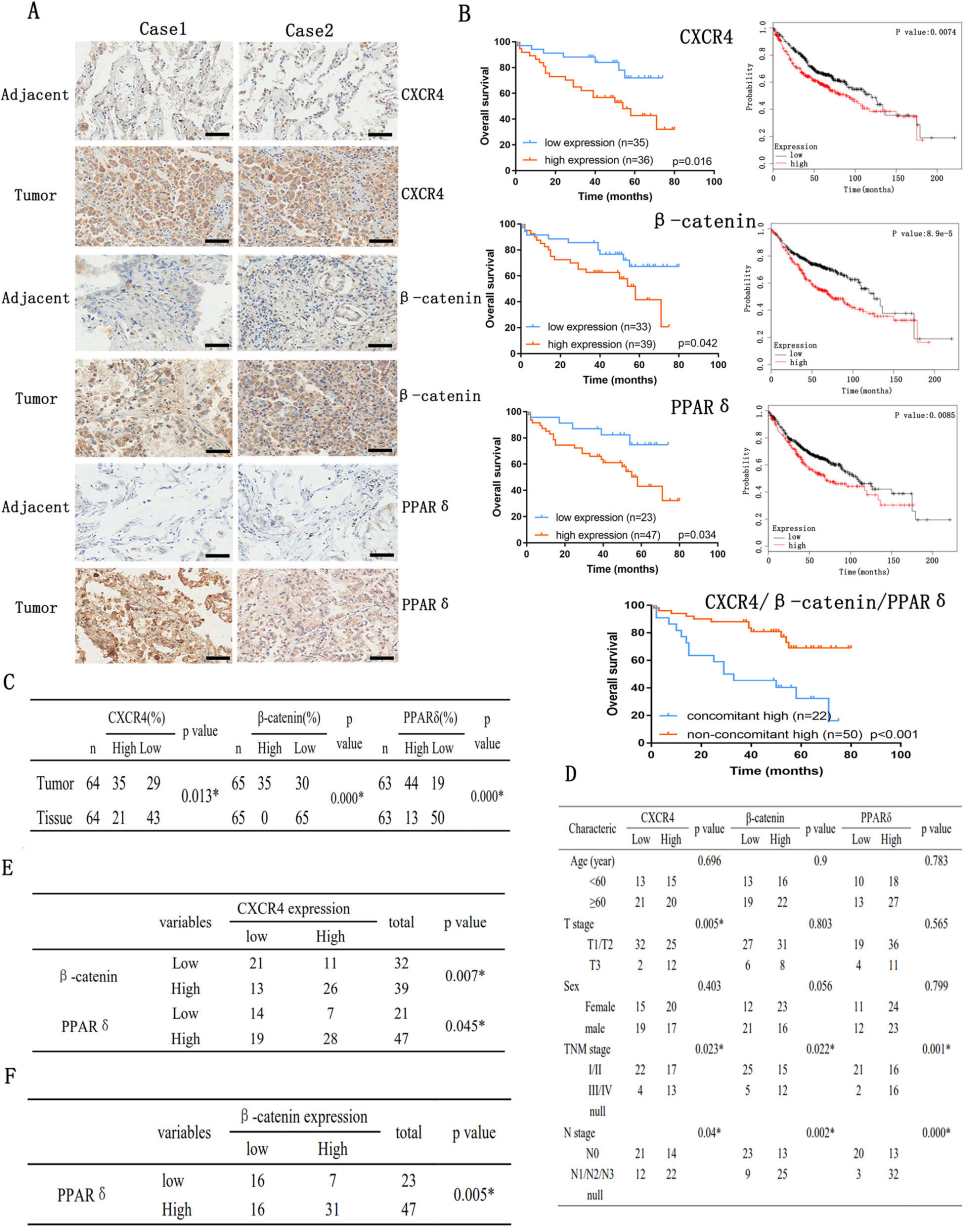
The high expression of CXCR4, β-catenin, and PPARδ is positively correlated and predicts poor prognosis in human lung ADC. **a**, **c** CXCR4, β-catenin, and PPARδ protein levels were evaluated in 75 human lung ADC tissues using immunohistochemical staining of tissue microarrays; scale bar: 100 μm. Case 1 and Case 2 represent two different patients. For each case, tumour and corresponding tissues were from one patient. **b** Kaplan–Meier analysis was used to evaluate patients’ overall survival with lung ADC and high or low expression of CXCR4, β-catenin, and PPARδ. **d** Analysis of the clinicopathological significance of CXCR4, β-catenin, and PPARδ protein in 75 human lung ADC tissues. **e**, **f** Pearson correlation analysis was performed to detect correlations between the expression of CXCR4, β-catenin, and PPARδ.

The correlation among CXCR4, β-catenin, and PPARδ expression was investigated in 75 human lung ADC tissues using immunohistochemical staining of tissue microarray. The analysis revealed that CXCR4, β-catenin, and PPARδ were all highly expressed in lung ADC tissues: CXCR4: > 54% (35/64 cases), β-catenin: > 53% (35/65 cases), and PPARδ: > 69% (44/63 cases) (Fig. 7a, c). The correlations between the expression of CXCR4, β-catenin, and PPARδ were also analysed using Pearson’s correlation analysis. CXCR4 was highly correlated with β-catenin (*p* = 0.007) and PPARδ (*p* = 0.045). β-catenin was also associated with PPARδ (*p* = 0.005; Fig. 7e, f).

After examining the clinicopathological significance of CXCR4, β-catenin, and PPARδ, the results showed that these three proteins are all associated with regional lymph node involvement (*p* = 0.04, *p* = 0.002, and *p* = 0.000, respectively) and tumour lymph node metastasis (TNM) staging (*p* = 0.023, *p* = 0.022, and *p* = 0.001, respectively; Fig. 7d).

Furthermore, Kaplan–Meier analyses of overall survival (OS) showed that the high expression group (immunohistochemical staining with strong and moderate expression) had poorer prognosis than that of the low expression group (immunohistochemical staining with weak and negative expression) (*p* < 0.01), and the concomitant high expression of CXCR4/β-catenin/PPARδ contributed to the poor prognosis in lung ADC patients (*p* < 0.001) (Fig. 7b). In addition, the relationship between the expression of the three proteins and OS in a 720 lung ADC patient cohort from the KMPLOT database (http://www.kmplot.com) was assessed using Kaplan–Meier analyses, which revealed that patients with low expression of all three proteins had significantly longer OS than their counterparts. These findings indicate a high incidence of CXCR4/β-catenin/PPARδ axis activation in lung ADC patients. Therefore, high migratory and invasion capabilities are characteristic of lung ADC cells.

## Discussion

CAFs have been reported to contribute to tumour invasiveness and metastasis by inducing an EMT phenotype^34,35^, although the role of CAFs in regulating EMT is not fully characterised. To address the gaps, we isolated CAFs from fresh human lung ADC clinical specimens and examined the effect of CAFs on lung ADC EMT. Based on the heterogeneity of CAF origin and function, CAFs affect tumour development differently, depending on the characteristics of cancer cells, tumour stage, and secreted factors. For example, Wnt signalling is known to contribute to tumour progression;^36^ however, Wnt3A from CAFs both promotes and inhibits development of patient-derived breast xenograft tumours^37^. These findings suggest that CAF populations derived from different types of carcinomas play specific roles. In this study, an effect on EMT of the lung ADC cell line was observed in the presence of the CAF-CM derived from patients with lung ADC. CAFs secrete different kinds of growth factors, cytokines, and proteases, including SDF-1, that directly act on tumour cells^38^. We performed SDF-1 ELISA analysis on the medium conditioned by each CAF population. The outcomes confirmed that the level of SDF-1 protein in the CAF-CM was increased in contrast to that produced with the corresponding NF population. Furthermore, the use of an SDF-1 neutralising antibody efficiently neutralised CAF-derived SDF-1 and reversed the EMT effect on lung ADC cells. These results suggest that CAFs from lung cancer clinical specimens exert an EMT-promoting effect via paracrine SDF-1 secretion in lung ADC.

CXCR4 is an evolutionarily highly conserved G protein-coupled receptor detected in multiple cancers, including lung ADC. SDF-1 is an essential ligand for CXCR4 and has been proven to affect CXCR4 expression and prompt EMT^39^. Lung ADC has been shown to be associated with an activated SDF-1/CXCR4-axis. However, so far, it has been found that SDF-1 has two receptors: CXCR4 and CXCR7; Hence, to clarify whether SDF-1 binding to CXCR4 induced lung cancer EMT, RNA interference experiments were carried out to knock down CXCR4 expression in A549 and SPCA-1 cells. Both normal and cancer fibroblasts responded well to SDF-1 stimulation (shown by increase in EMT and invasiveness), whereas the *CXCR4* knocked down cells did not significantly respond to the SDF-1. In addition, tissue microarray was used to verify that CXCR4 was overexpressed in the lungs of ADC patients and was positively correlated with poor prognoses. Hence, activation of the SDF-1/CXCR4-axis supports lung EMT and invasion.

β-catenin is an intracellular scaffold protein that interacts with adhesion molecules, transmembrane‑type mucins, signalling regulators, and epigenetic or transcriptional regulators. It is generally known that β-catenin signalling is involved in tumour EMT and metastasis. Recent research has shown that the SDF-1/CXCR4 may additionally promote tumour EMT by way of activating the β-catenin signalling pathway in breast and colon most cancers^20,21^. However, the associate mechanism in lung ADC remains unknown. Therefore, we examined the relationship between SDF-1/CXCR4 and β-catenin activation in lung ADCs. β-catenin activity was quantified by Luciferase reporter assay and active β-catenin was found to translocate from the cell membrane to nucleus. However, these effects were inhibited by treatment with β-catenin inhibitors. These results indicate that β-catenin signalling is abnormally activated in SDF-1-mediated EMT in A549 and SPCA-1 cells. The expression of CXCR4 and β-catenin was further evaluated to analyse signal transduction in SDF-1 mediated lung ADCs. Similar to what was reported for colon cancer^40^, inhibition of CXCR4 inhibited the nuclear expression and nuclear localisation of β-catenin even after treating lung ADC cells with SDF-1. However, β-catenin inhibitors did not impact CXCR4 expression, indicating that β-catenin may also be a downstream signalling molecule in the CXCR4 pathway. This is confirmed by the tissue microarray results, which showed that β-catenin was upregulated and that its expression correlated positively with CXCR4 expression in lung ADC tissues. Hence, we speculate that SDF-1/CXCR4/β-catenin signalling in lung ADC is a critical determinant of its metastatic potential.

PPARδ was identified as a potential downstream effector of β-catenin using bioinformatics analysis. To date, the role of PPARδ in cancer metastasis is not clear and is highly debated. For example, PPARδ in colorectal cells drastically affects EMT, migration, and invasion, and promotes metastasis^41^. However, others have reported that the upregulated PPARδ reduces cell invasion in vitro in human pancreatic ductal carcinoma^42^. These contradictory findings suggest that PPARδ differentially affects tumour-associated functions depending on the cell type, cellular context, stage of differentiation, and the environment of soluble mediators. In the present study, we observed that PPARδ was induced by SDF-1 and stimulated the EMT of lung ADC cells. Meanwhile, inhibition of β-catenin significantly downregulated PPARδ expression, although inhibition of PPARδ did not interrupt β-catenin signalling. These findings suggest that PPARδ is a downstream regulator. So far, the specific mechanism between β-catenin and PPARδ remains unclear, although our ChIP assays showed β-catenin binding to the PPARδ promoter. So, in the following work, we will further investigate the mechanism to address this knowledge gap.

Taken together, we identified CXCR4/β-catenin/PPARδ signalling in the CAF-induced SDF-1-mediated regulation of EMT in lung ADC. These findings provide insights into the process by which EMT in lung ADC is regulated in the tumour microenvironment and reveal a potential target that can be utilised in the production of precise therapeutic drugs for lung ADC.

## Supplementary information

Supplementary Figure legends

Supplementary Figure 1.

Supplementary Figure 2.

Supplementary Figure 3.
