# Correction: Cancer-associated fibroblast-derived SDF-1 induces epithelial-mesenchymal transition of lung adenocarcinoma via CXCR4/β-catenin/PPARδ signalling

**DOI:** 10.1038/s41419-025-07368-8

**Published:** 2025-02-27

**Authors:** Yingyan Wang, Wen Lan, Mingxin Xu, Jing Song, Jun Mao, Chunyan Li, Xiaohui Du, Yunling Jiang, Encheng Li, Rui Zhang, Qi Wang

**Affiliations:** 1 Department of Respiratory Medicine, The Second Affiliated Hospital, Dalian Medical University, No. 467 Zhongshan Road, Dalian, 116023 Liaoning Province China; 2 Laboratory Center for Diagnostics, Dalian Medical University, No. 9 West Section Lvshun South Road, Dalian, 116044 Liaoning Province China; 3 Department of Respiratory Medicine, Ganzhou Municipal Hospital, No. 49 Dagong Road Zhanggong district, Ganzhou, 341000 Jiangxi Province China; 4 Department of Pathology, Dalian Medical University, No. 9 West Section Lvshun South Road, Dalian, 116044 Liaoning Province China; 5 Department of Gastroenterology, The First Affiliated Hospital, Dalian Medical University, No. 222 Zhongshan Road, Dalian, 116011 Liaoning Province China; 6 Department of Scientific Research Center, The Second Affiliated Hospital, Dalian Medical University, No. 467 Zhongshan Road, Dalian, 116023 Liaoning Province China

Correction to: *Cell Death and Disease* 10.1038/s41419-021-03509-x, published online 26 February 2021

In this article Fig. 6C showed a lot of band results of Western blotting assays, that have been mistakenly re-used in the GAPDH band of the A549 group as an internal reference Histone for SPCA-1 nuclear proteins and further quantified the relative levels of β-catenin.

Different internal references may lead to different analysis results. So, we traced back the original bands and found the results of three independent replicate experiments performed to detect the expression of β-catenin and corresponding Histone after nuclear protein extraction in SPCA-1 cells. The relative levels of β-catenin were also re-quantified.

Fortunately, this correction did not affect the statistical differences in the levels of β-catenin between groups.

Correct Fig. 6
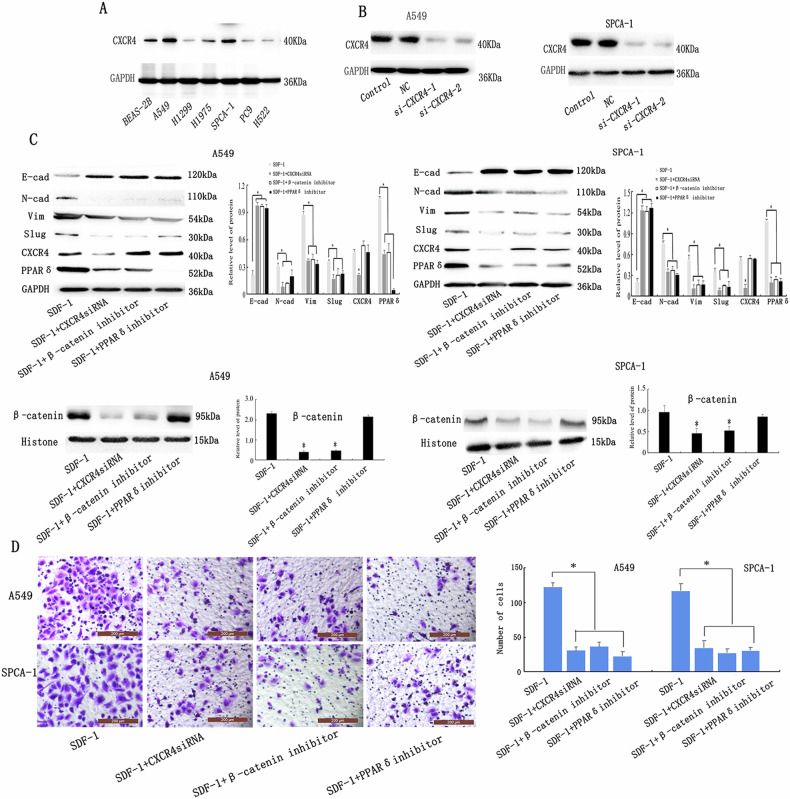


Incorrect Fig. 6
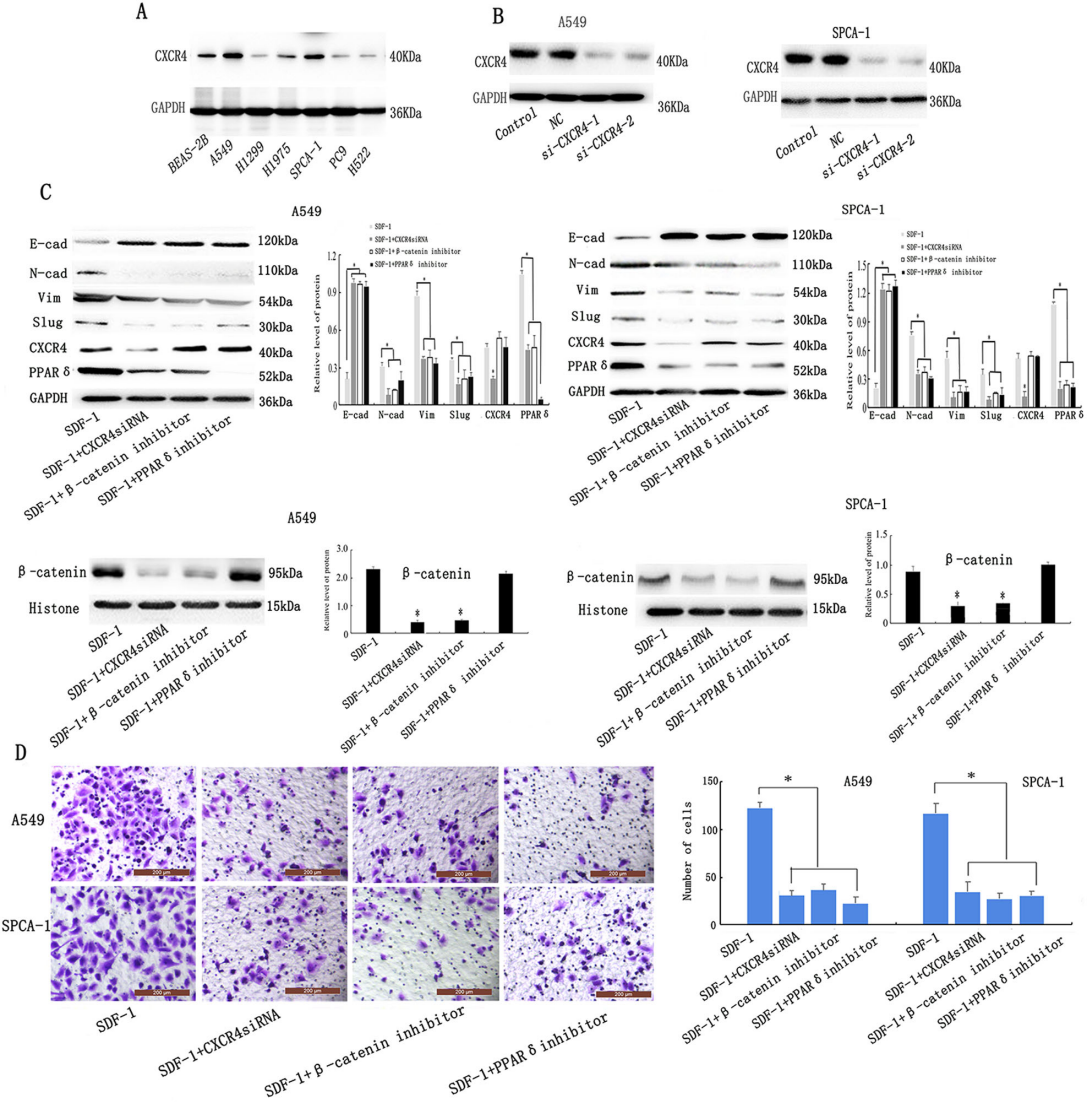


The original article has been corrected.

