# Supplementary material for: Cancer-associated fibroblast-derived SDF-1 induces epithelial-mesenchymal transition of lung adenocarcinoma via CXCR4/β-catenin/PPARδ signalling
Source: Cell Death Dis. 2021 Feb 26;12(2):214. doi: 10.1038/s41419-021-03509-x (PMC7910618; doi:10.1038/s41419-021-03509-x)
Supplement: Supplementary file 1 — Supplementary Figure legends [file 41419_2021_3509_MOESM1_ESM.docx]

**Supplementary Figure 1.** β*-*catenin is activited by the SDF-1/CXCR4 axis in A549 and SPCA-1 cells. (A) Localization of β-catenin was analyzed by immunofluorescent staining in control cells，CAF-CM cells, CAF-CM+CXCR4siRNA cells and CAF-CM+anti-SDF-1Ab cells. Nuclei were stained with DAPI (blue). β-catenin were indicated by red label. Scale bar: 25μm. (B) The reporter construct expressing luciferase gene under control of TCF/Lef was introduced into cells, luciferase activity was measured in 3 experiments; average data are presented (Mean±SD, * p<0.05).

**Supplementary Figure 2.** The PPARδ expression in A549 and SPCA-1 cells treated with CAF-CM , CAF-CM+the β-catenin inhibitor and CAF-CM+ anti-SDF-1 Ab. *P < 0.05,

**Supplementary Figure 3.** ChIP assays is performed to examine β-catenin binding to the PPARδ promoter. **P < 0.01.
