# Supplementary figures and images for: Cancer-associated fibroblast-derived SDF-1 induces epithelial-mesenchymal transition of lung adenocarcinoma via CXCR4/β-catenin/PPARδ signalling
Source: Cell Death Dis. 2021 Feb 26;12(2):214. doi: 10.1038/s41419-021-03509-x (PMC7910618; doi:10.1038/s41419-021-03509-x)

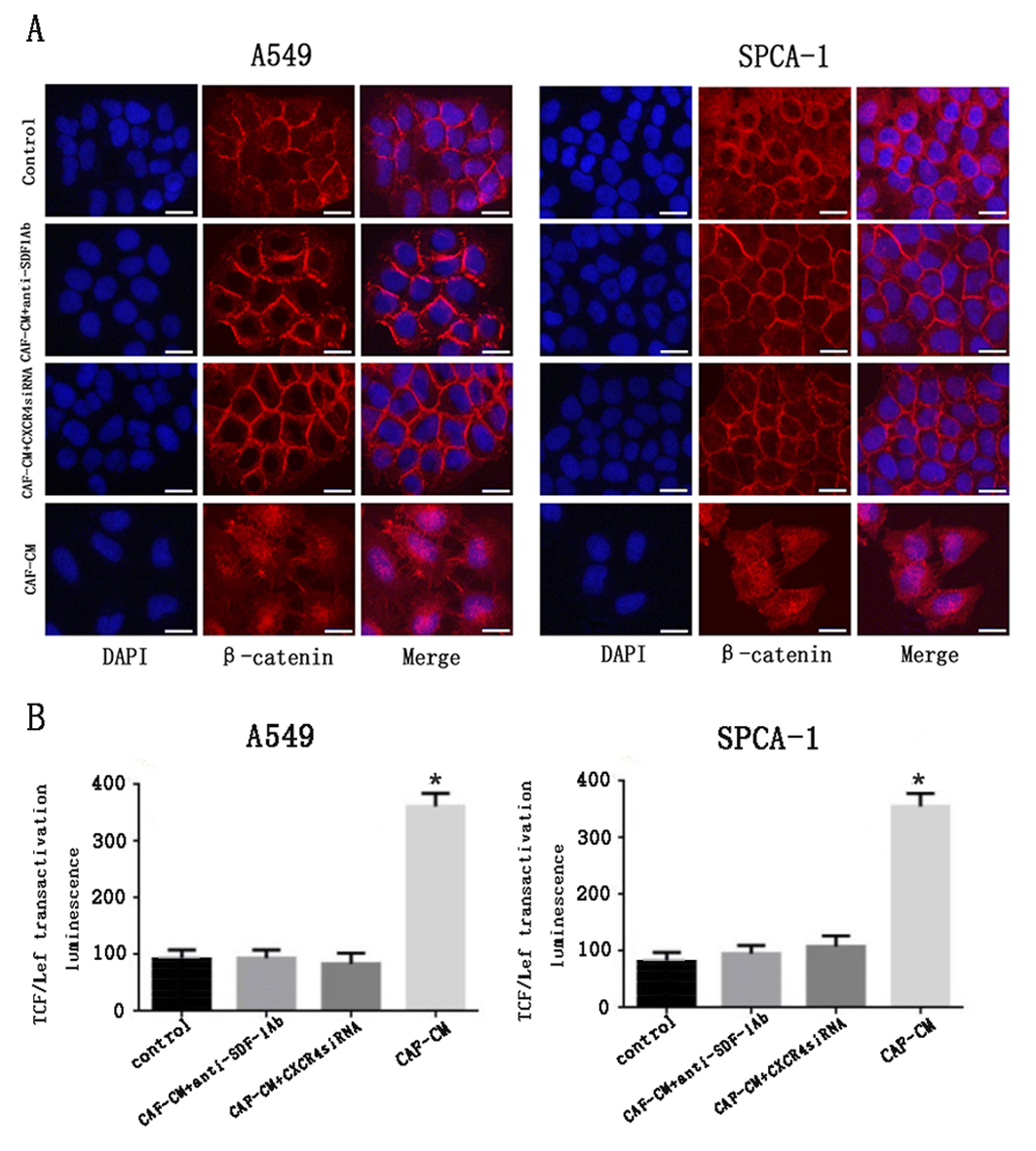

Supplement: Supplementary file 2 — Supplementary Figure 1. [file 41419_2021_3509_MOESM2_ESM.png]

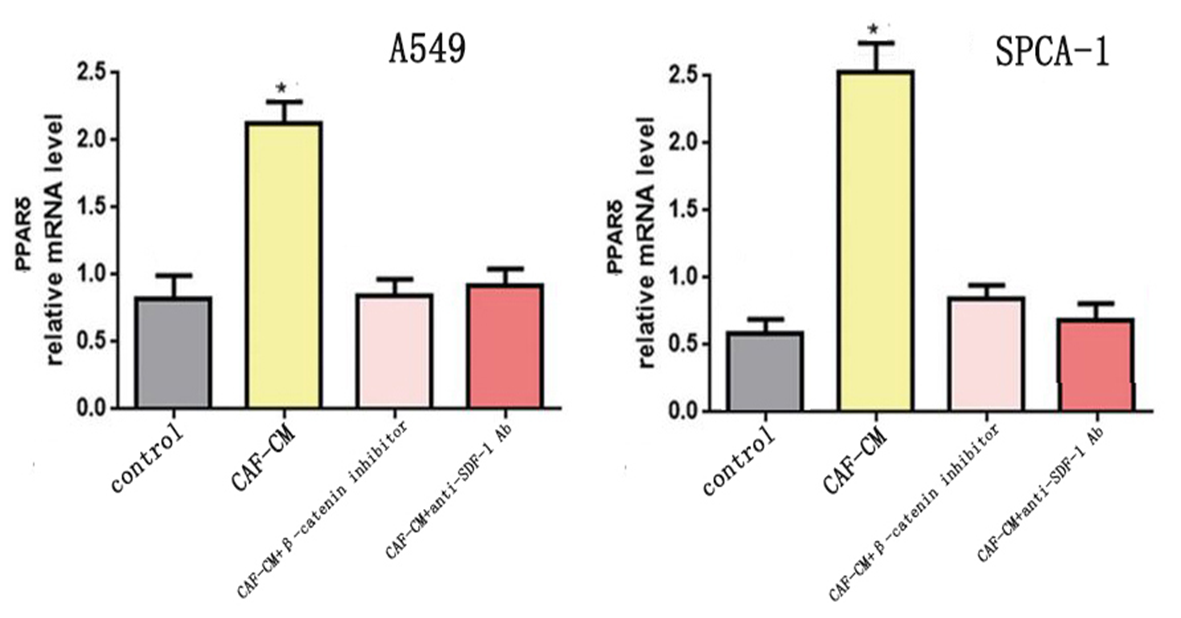

Supplement: Supplementary file 3 — Supplementary Figure 2. [file 41419_2021_3509_MOESM3_ESM.png]

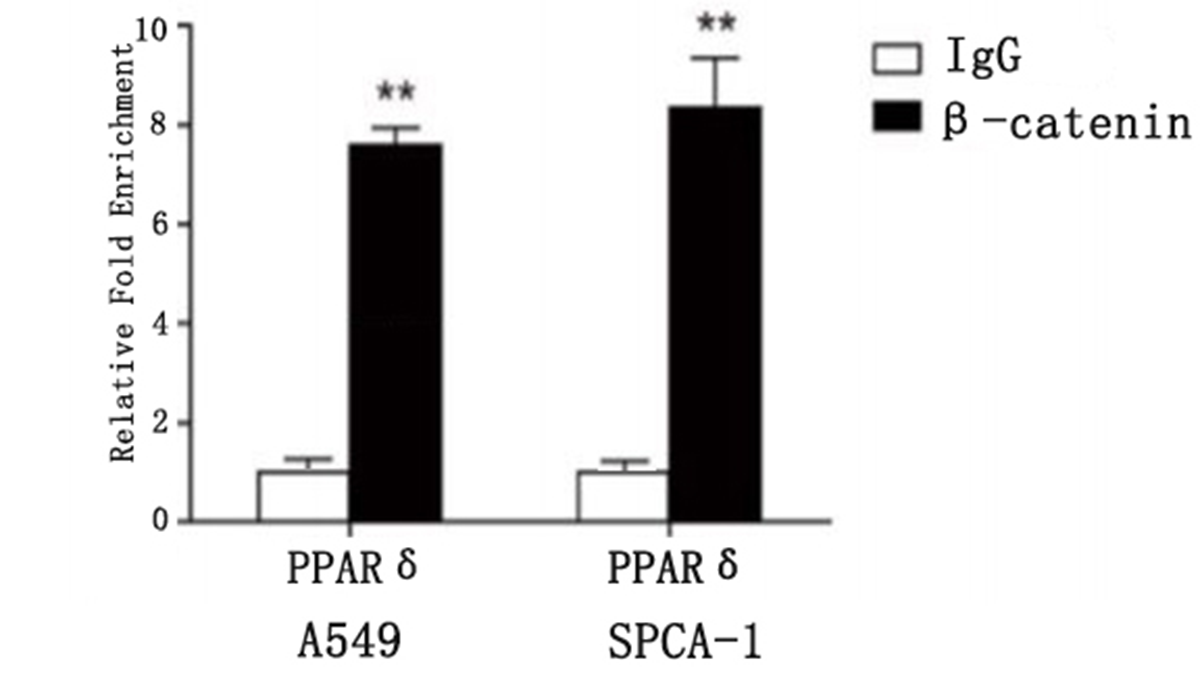

Supplement: Supplementary file 4 — Supplementary Figure 3. [file 41419_2021_3509_MOESM4_ESM.png]
